# Pros and Cons of Denosumab Treatment for Osteoporosis and Implication for RANKL Aptamer Therapy

**DOI:** 10.3389/fcell.2020.00325

**Published:** 2020-05-14

**Authors:** Ning Zhang, Zong-Kang Zhang, Yuanyuan Yu, Zhenjian Zhuo, Ge Zhang, Bao-Ting Zhang

**Affiliations:** ^1^School of Chinese Medicine, Faculty of Medicine, The Chinese University of Hong Kong, Hong Kong, China; ^2^Law Sau Fai Institute for Advancing Translational Medicine in Bone and Joint Diseases, School of Chinese Medicine, Hong Kong Baptist University, Hong Kong, China

**Keywords:** osteoporosis, denosumab, RANKL, aptamer, SELEX

## Abstract

Osteoporosis is age-related deterioration in bone mass and micro-architecture. Denosumab is a novel human monoclonal antibody for osteoporosis. It is a receptor activator of nuclear factor-κB ligand (RANKL) inhibitor, which binds to and inhibits osteoblast-produced RANKL, in turn reduces the binding between RANKL and osteoclast receptor RANK, therefore decreases osteoclast-mediated bone resorption and turnover. However, adverse events have also been reported after denosumab treatment, including skin eczema, flatulence, cellulitis and osteonecrosis of the jaw (ONJ). Extensive researches on the mechanism of adverse reactions caused by denosumab have been conducted and may provide new insights into developing new RANKL inhibitors that achieve better specificity and safety. Aptamers are single-stranded oligonucleotides that can bind to target molecules with high specificity and affinity. They are screened from large single-stranded synthetic oligonucleotides and enriched by a technology named SELEX (systematic evolution of ligands by exponential enrichment). With extra advantages such as high stability, low immunogenicity and easy production over antibodies, aptamers are hypothesized to be promising candidates for therapeutic drugs targeting RANKL to counteract osteoporosis. In this review, we focus on the pros and cons of denosumab treatment in osteoporosis and the implication for novel aptamer treatment.

## Introduction

Osteoporosis is a disease in which the microstructure of bone deteriorates, bone mass is low, and bone fragility increases, therefore the risk of fracture increases in affected people (Black and Rosen, 2016; Eastell et al., 2016). Due to bone metabolism disorders caused by the rapid decline of estrogen levels in the body, osteoporosis is most common in postmenopausal women. Increasing bone turnover and reduced bone density result in severe bone loss and ultimately develop into osteoporosis (Deeks, 2018). The number of people who suffered from osteoporosis was approximately two hundred million in the world (Sözen et al., 2017). The female to male ratio is 80 to 20%, while it was reported that male patients developed other clinical complications related to osteoporosis (Alswat, 2017). As a common symptom of osteoporosis, osteoporotic fractures can result in further disability and early mortality. Osteoporotic fractures occur most frequently in the vertebrae (spine), carpals, hips, pelvis and upper arms (Warriner et al., 2011). Many patients require long-term nursing home care, which would leave a huge burden on the patient’s family and society. Total costs of treatment for osteoporotic fractures were estimated to be 17 billion in 2003 in the United States, and the figure is predicted to surpass 25 billion by the year 2025 (Cauley, 2013). Therefore, a lot of in-depth researches have been conducted to reveal the underlying mechanisms of osteoporosis and identify the potential therapeutic targets.

Bone morphogenesis and remodeling depend on the combined activities of osteoblasts-mediated bone formation and osteoclasts-mediated bone resorption (Zhang et al., 2014). The process is a dynamic process throughout the entire life of the individual, and plenty of systemic and local regulators of osteocytes are involved in bone remodeling (Miller, 2009). This mechanism not only protects the integrity of the bones but also makes the bones an adequate reservoir of calcium and phosphorus. According to the functions of osteoblasts and osteoclasts, bone remodeling can be divided into three primary stages. The initial stage is the differentiation of osteoclast precursor cells, activation of osteoclasts and bone resorption. Osteoclast apoptosis occurs in the intermediate stage and the meantime, osteoblasts accumulate and differentiate to form new bone in the bone lacuna. During the final stage, bone resorption is completed through osteogenesis and mineralization (Lemaire et al., 2004). Even in healthy bodies, osteoclasts-mediated bone resorption takes only a few weeks, while osteoblasts-mediated bone formation takes months (Sims and Martin, 2014). Therefore, the key to preventing bone loss is to inhibit the function of osteoclasts. The control and regulation of osteoclast activation and differentiation is achieved by a family of biologically related TNF receptor (TNFR)/TNF-like proteins: osteoprotegerin (OPG), the receptor activator of NF-κB (RANK), and RANK ligand (RANKL) (Lacey et al., 1998; Faienza et al., 2018).

Early animal experiments and clinical trials show that RANKL-targeted therapies have the potential to treat diseases relevant to osteoclast-mediated bone loss. RANKL inhibition results in a decrease in osteoclastogenesis and osteoclast activity, thereby reducing bone resorption (Bekker et al., 2004). Denosumab, a targeted antibody that binds and inhibits RANKL to reduce bone resorption, is approved by the U.S. Food and Drug Administration (FDA) for the treatment of osteoporosis in postmenopausal (PM) women with a high fracture risk (Dore, 2011; Scott and Muir, 2011). The European Medicines Agency has also declared the marketing authorization valid of denosumab for the treatment of osteoporosis in PM women at increased risk of fractures (European Medicines Agency, 2013). Apart from antibodies, some agents targeting RANKL have also been investigated to interact with RANKL specifically and interfere with its interaction with RANK (Sisay et al., 2017). Aptamers, small single-stranded oligonucleotides, are capable of binding target molecules with high affinity and specificity (Burnett and Rossi, 2012; Song et al., 2012). They are nucleotide analogs of antibodies, but it is much easier and cheaper to generate aptamers than antibodies (Kulbachinskiy, 2007; Lakhin et al., 2013). Moreover, aptamers are neither immunogenic nor toxic (Bouchard et al., 2010). Aptamer-targeted therapeutic drugs have been used in the treatment of various cancers, macular degeneration, vascular hemophilia and diabetes, which have shown excellent application prospects in the field of biomedicine (Yang et al., 2011; Cibiel et al., 2012).

This review focuses on the advantages and disadvantages of the use of the anti-RANKL antibody, denosumab, in the treatment of PM osteoporosis. Besides, the prospect and challenge of aptamers targeting RANKL as a therapeutic strategy for osteoporosis are discussed.

## RANKL as a Promising Therapeutic Target for Osteoporosis

**FIGURE 1:**
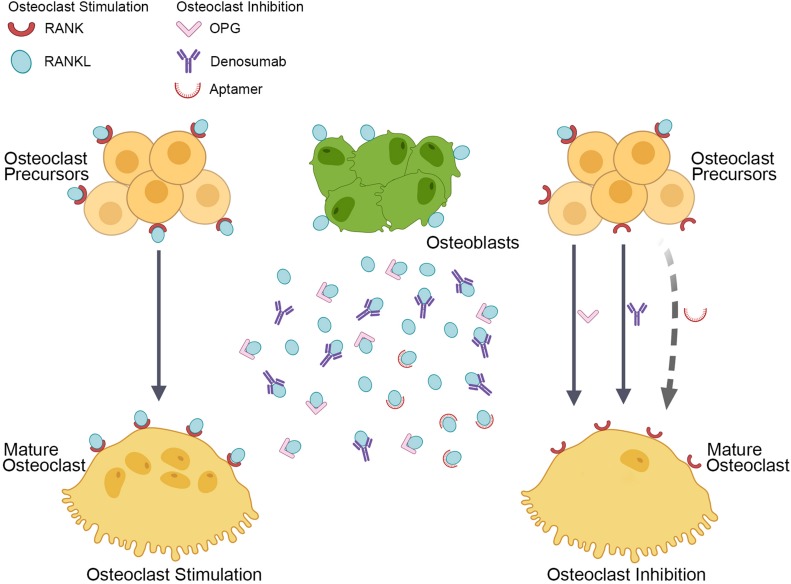
Current and hypothetical RANKL inhibitors to suppress osteoclastogenesis. Osteoblasts produce RANKL that binds its receptor, RANK, which is located on the surface of osteoclasts and their precursors. This binding stimulates the differentiation of preosteoclasts into multinucleated and mature osteoclasts (as shown by the solid arrow in the left). The process is prevented naturally by OPG and pharmacologically by denosumab, thereby inhibiting osteoclast formation, activity, and survival (as shown by solid arrows in the right). We hypothesized a RANKL inhibitor, the aptamer, which may specifically target RANKL and inhibit osteoclastogenesis (as shown by the dotted arrow in the right).

Receptor activator of nuclear factor-κB ligand is one of the tumor necrosis factor (TNF) superfamily members, and it is up-regulated in postmenopausal women, which can be modified mainly by estrogen supplementation (Eghbali-Fatourechi et al., 2003). RANKL is a homotrimeric transmembrane protein expressed by osteocytes, macrophages, osteoblasts, bone marrow stem cells and activated T lymphocytes (Lacey et al., 2012; Mori et al., 2013). It owns a carboxy-terminal domain that is homologous to TNF, and it is expressed on the cell surface of osteoblasts, resulting in cell-to-cell-dependent contact with osteoclast precursor cells to promote the formation of osteoclasts. RANKL also inhibits osteoclast apoptosis. RANK, the signaling receptor of RANKL, is expressed on the surface of osteoclast precursor cells, mature osteoclasts, dendritic cells and other tumor cells. It initiates osteoclastogenesis (Figure 1) by combining with carboxy-terminal domains of RANKL to trigger intracellular signaling cascades, which causes osteoclast precursors to evolve into multinucleated cells and differentiate into mature osteoclasts (Zaidi et al., 2003; Park et al., 2017).

Osteoprotegerin, a soluble decoy receptor, competitively binds to the trimer RANKL and negatively regulates the interaction of RANKL-RANK, thereby inhibiting the differentiation of osteoclast precursors (Figure 1) and inducing osteoclast apoptosis (Simonet et al., 1997). It is a soluble secreted protein lacking a transmembrane domain and a cytoplasmic domain, principally expressed by osteoblasts, bone marrow stromal cells, mature B cells and macrophages, and regulating the critical balance between bone formation and bone resorption (Yasuda et al., 1998). The amino-terminus of OPG owns a cysteine-rich region, while the carboxy-terminus forms a dimer that competitively binds to the trimer RANKL, resulting in preventing RANKL-induced generation and maturation of osteoclasts. It has been found that OPG binds RANKL approximately 500 times higher affinity than RANK (Infante et al., 2019). OPG-knockout mice resulted in a significant reduction in bone mass, while overexpression of OPG leads to a decrease in the number of osteoclasts and a noticeable increase in bone mass (Nakamura et al., 2003). Therefore, the OPG/RANKL ratio is closely related to osteoclast formation and ultimately affects bone density and bone strength. Genetic mutations in the human RANKL gene and RANKL knockout mice have been associated with osteoclasts deficiency and severe osteosclerosis (Sobacchi et al., 2007; Crockett et al., 2011).

In summary, RANKL plays a vital role in the process of osteoclast differentiation. Besides, RANKL can also activate mature osteoclasts, prolong their survival time and enhance bone resorption capacity. Since the use of related antibodies, peptides and natural compounds to inhibit RANKL can prevent the formation and function of osteoclasts, RANKL is regarded as a potential target for the treatment of osteoporosis.

## Current Therapeutic Strategies Targeting RANKL

At present, it has been found that a variety of natural and synthetic drugs can affect the function of osteoclasts through the OPG/RANK/RANKL signaling pathway, such as recombinant human osteoprotegerin, strontium compounds and human RANKL monoclonal antibodies.

Recombinant human OPG (rhOPG) was the first RANKL inhibitor that has been examined in humans (Dempster et al., 2012). It is a modified natural OPG, which makes it more biologically active and own a longer half-life. Studies have shown that rhOPG can significantly inhibit osteoclast activity and reduce bone resorption, resulting in accelerating bone maturation and increasing bone mineral density (BMD). A clinical trial demonstrated the validity of RANKL antagonization to reduce bone turnover in humans by subcutaneous injections of rhOPG (Bekker et al., 2001). RhOPG is reported to prevent bone loss diseases and participate in inhibiting alveolar bone absorption during periodontitis (Jin et al., 2007). Nonetheless, long-term medication may cause immune responses and safety concerns due to its massive molecular weight.

The trace element strontium has the dual effects of preventing bone resorption and promoting bone formation. Studies have shown that strontium mainly functions through the OPG/RANK/RANKL signaling pathway, resulting in decreased bone resorption (Mi et al., 2017). Strontium ranelate is a representative drug of strontium compounds, and it contains two strontium atoms (Cianferotti et al., 2013). The apparent increase of OPG plays a vital role in the treatment of osteoporosis with strontium ranelate and could be used as a convincing measurement to examine the therapeutic effect.

Another therapeutic strategy that explicitly targets RANKL is the use of an anti-RANKL antibody. Denosumab, a fully human monoclonal IgG2 antibody, binds with RANKL selectively and displays high affinity, which binds and neutralizes the activity of human RANKL to inhibit bone resorption and may also prevent progression of bone erosion (Kostenuik et al., 2009). At a quite low concentration, denosumab can reduce the bone rebuilding cycle, inhibit the action of osteoclasts, and increase bone density and strength. The effect of denosumab treatment on fracture prevention in women with postmenopausal osteoporosis was evaluated in an international, multicenter, randomized, double-blind, and placebo-controlled study known as Fracture Reduction Evaluation of Denosumab in Osteoporosis Every 6 Months (FREEDOM) (Cummings et al., 2009). Denosumab was effective in reducing fracture risk in PM women with osteoporosis in the FREEDOM study.

## Denosumab: Clinical Data and Efficacy Evaluation

Denosumab is the first and only approved antagonist targeting RANKL, which can effectively block the interaction between RANKL and RANK, thereby inhibiting the formation of osteoclasts and enhancing bone strength. Denosumab could be administered once in 6 months by a subcutaneous injection of 60 mg each time. Large-scale clinical trials have shown that it can significantly improve bone turnover markers (BTM). Bone-resorption markers of collagen telopeptide of type 1 collagen (CTX) and procollagen type 1 N-terminal propeptide (PINP) were significantly reduced, while the BMD of the lumbar spine and hip joints was increased after Denosumab treatment (Cummings et al., 2009; Eastell et al., 2011). The FREEDOM study included 7,868 postmenopausal osteoporosis patients in clinical trials. According to the clinical data, denosumab significantly eliminated the risk of new vertebral fracture by 68%, non-vertebral fracture by 20% and hip fracture by 40% relative to placebo across 3 years (Cummings et al., 2009).

Similarly, another research reported that the denosumab group has a significant lower incidence of vertebral fractures and low-energy non-vertebral fractures compared to the placebo group, regardless of conditions like ages, previous fracture status and other anti-osteoporotic treatments (Palacios et al., 2015). A 7-year long-term study found that the effect of denosumab on increasing bone density had sustained through years. Patients who received long-term treatment of denosumab maintained a low incidence of fractures and reduced overall and non-vertebral fracture rates by 38 and 46% compared to placebo (Bone et al., 2017). At the end of the research, the BMD of the long-term group (up to 10 years of denosumab treatment) substantially increased from the original baseline. The increments of each site were better than those of the 7-year treatment groups (e.g., lumbar BMD increased by 21.7 and 16.5%) (Bone et al., 2017). Furthermore, bone strength increased consistently with BMD at various sites under denosumab treatment (Simon et al., 2013; Zebaze et al., 2016).

The increase in bone density could be indirectly reflected in the morphology of bone cortex and cancellous bone, which showed instant improvement. A computed tomography scanning showed that denosumab reduced the cortical porosity of patients and increased hip joint bone strength by 7.9% (Zebaze et al., 2016). The FREEDOM trial confirmed the effect of denosumab on reducing fracture rates and increasing bone density. Compared with placebo, denosumab significantly increased BMD in the lumbar spine, hip joint, femoral neck, femoral tuberosity, and distal radius 1 month after treatment, and the effect continued to increase during the subsequent 36 months. A series of controlled clinical trials showed that denosumab possessed advantages over bisphosphonates in promoting bone density in postmenopausal patients with low bone mass or osteoporosis. Denosumab has shown a gradual upward trend and long-term potential for the treatment of osteoporosis and other bone metabolic diseases (Miller et al., 2016; Anastasilakis et al., 2018). Despite numerous advantages of denosumab, it has only been on the market in recent years, and its clinical efficacy remains to be scrutinized.

## The Potential Problems Related to the anti-RANKL Monoclonal Antibody, Denosumab

One of the disadvantages related to denosumab is that there are some adverse reactions after medication. Since RANKL is also abundantly expressed by dendritic cells and activated T lymphocytes, the antagonistic effect caused by denosumab could affect the immune system and result in individual risk of adverse events (Walsh and Choi, 2014). In the FREEDOM study, the adverse events in the denosumab treatment group included skin eczema (3%), flatulence (2.2%), and cellulitis (0.3%) (Cummings et al., 2009; Anastasilakis et al., 2012). Although the overall incidence of adverse events in the denosumab treatment group was close to that in the placebo group, some numerical imbalances showed that the majority of adverse events was related to specific ones, including skin infections (Watts et al., 2012). Denosumab could amplify skin allergies and inflammatory responses rather than increase susceptibility to infection (Ferrari-Lacraz and Ferrari, 2011). Since the interaction between RANK and RANKL was also essential in the development of immunocytes (Anastasilakis et al., 2012), the effect of denosumab of immunogenicity should be further investigated during clinical trial, even though the higher incidence of adverse events has not been discovered thus far during long-term (7–10 years) treatment of denosumab (Bone et al., 2017).

Patients treated with denosumab are also at risk of osteonecrosis of the jaw (ONJ). A systematic review suggested that 1.7% of patients suffered ONJ during tumor treatment with denosumab (Boquete-Castro et al., 2016). A study focusing on denosumab treatment in newly diagnosed multiple myeloma patients showed that the incidence of ONJ was higher in denosumab treatment groups than in earlier solid tumor bone-related events (Morgan et al., 2010), indicating that the higher incidence might be related to more prolonged exposure to the therapies in the trial (Raje et al., 2018). Therefore, those who have undergone invasive dental surgeries, installed dentures or other implants, and have poor oral hygiene should be alerted to the risk of ONJ and are strongly advised to receive clinical examinations regularly during medication (Boquete-Castro et al., 2016). When taking denosumab, those who are already having ONJ or who are most likely to develop ONJ should necessarily be prescribed by a dentist. Excessive dental surgery may worsen the situation. Therefore, the benefit-risk assessment can be performed according to the patient’s conditions to determine whether denosumab needs to be discontinued.

Since denosumab forcefully inhibited bone turnover, serum calcium levels were evaluated carefully in FREEDOM (Dempster et al., 2012). Although the overall incidence of hypocalcemia during denosumab treatment is low (Jamal et al., 2011), for patients with renal insufficiency, blood calcium levels should be monitored and corrected during treatment. Simultaneously, an adequate supplement of calcium and vitamin D is recommended (Body et al., 2015). Other studies have shown that despite calcium supplements and calcitriol, approximately 14% of patients developed varying degrees of hypocalcemia within 6 months under denosumab treatment (Huynh et al., 2016). Severe renal insufficiency is one of the dominating risk factors.

While the mechanisms involved in the adverse effects of denosumab remains unknown so far, further research of the pathogenesis may give insights into the accurate selection of next-generation inhibitors.

## Aptamer: a Promising Therapeutic Strategy for Osteoporosis

Although the therapeutic efficacy of denosumab has been studied widely and thoroughly, the benefit/risk profile of denosumab still needs to be further studied in clinical trials. Other alternative agents targeting RANKL with low-cost and high safety for the treatment of osteoporosis and other bone-related diseases have attracted a lot of attention. Among all of these potential agents, aptamers are promising candidates due to their unique properties and promising applications.

Aptamers are small single-stranded oligonucleotides, which could specifically bind with target molecules. Aptamers are screened from oligonucleotide libraries, which generally consists of a fixed sequence at both ends of the oligonucleotide chain and a random sequence with a length of 20–60 bp in the middle, using a gold-standard methodology named SELEX (systemic evolution of ligands by exponential enrichment) (Blank and Blind, 2005). Since there are a large number of permutations and combinations of nucleotides in the intermediate sequence, different spatial conformations can be formed, thereby nearly all of the spatial structures in nature that may interact with target molecules are simulated (Tsao et al., 2017).

A comparison between aptamers and antibodies exhibited distinct advantages of aptamers. Compared to antibodies, (i) aptamers are prepared through *in vitro* screening and can be produced using cell-free chemical synthesis (ii), aptamers are not immunogenic and can be used for *in vivo* diagnosis or treatment (iii) they are smaller than antibodies and can be used for intracellular diagnosis and treatment and (iv) chemically synthesized aptamers own high accuracy, reliable repeatability, and few variations between batches in production (Song et al., 2012). Reporter genes such as fluorescein or biotin can be accurately combined with aptamers at specific sites for the research interests (Reverdatto et al., 2015). In summary, aptamers have multiple advantages over antibodies and can be promising candidates for novel therapeutic strategies for various diseases.

To date, the U.S. FDA has approved an aptamer-based drug called Mucagen, and the other ten aptamers have been studied in clinical trials (Zhou and Rossi, 2017), which demonstrates that aptamers can also be used directly as drugs. Clinical studies of Mucagen treatment in age-related macular degeneration (AMD) patients have exhibited stabilization or improvement of vision in 80% of patients at 3 months without any toxicity (Vinores, 2006). No treatment-related side effects were noted in previous phases of clinical trials, while phase III clinical trials showed endophthalmitis occurred in 1.3% of patients, traumatic injury to the lens in 0.7%, and retinal detachment in 0.6%, accounting for the most severe adverse effects (Gragoudas et al., 2004). Collectively, Mucagen has maintained an affirmative safety profile with only occasional adverse events. Other ten aptamers have undergone clinical trials for the treatment of various conditions, including macular degeneration, coagulation, oncology, and inflammation (Zhou and Rossi, 2017). Most of them have exhibited positive efficacy and non-toxicity except one aptamer, Spiegelmer, which interferes with tumor proliferation and metastasis for cancer therapy (Roccaro et al., 2014). In the phase I clinical study of Spiegelmer for multiple myeloma treatment, several mild adverse events have been reported, including headache, nasopharyngitis, contusion and rhinitis (Vater et al., 2013). A subsequent phase II clinical trial of Spiegelmer and combination with bortezomib-dexamethasone reported adverse events of thrombocytopenia, anemia, and diarrhea (Ludwig et al., 2017). Notably, the intensities of all of the adverse events were mild and no severe adverse events were reported. Nonetheless, the safety and tolerability of aptamers are still under evaluation in the following phases of clinical trials (Kaur et al., 2018). Up to now, all of the aptamers that undergone clinical trials function as antagonists, while aptamers could also act as agonists that activate target receptors and carriers that delivering drugs to target molecules and proteins (Zhou and Rossi, 2017).

Taken together, the aptamer has the potential to be the therapeutic agent targeting RANKL to counteract osteoporosis.

## Aptamer Targeting RANKL: Hypothetical Point and the Technical Aspect

The standard methodology for aptamer selection, known as SELEX, can be separated into two alternating stages. The technology firstly designs and artificially synthesizes a random single-stranded oligonucleotide library. There is a random sequence with a length of 20 to 60 bp in the middle of the oligonucleotide chain flanked by fixed sequences of 20 to 40 bp at both ends. T7 RNA polymerase promoter sequence is added to the 5′ end, and a pair of corresponding primers are designed to amplify the original oligonucleotides by a polymerase chain reaction (PCR). In the second stage, the original synthesized library is incubated with target molecules and then filtered to isolate target molecule-nucleic acid complexes. The interacting oligonucleotides are eluted to perform PCR amplification to obtain a sub-library, which is subjected to the subsequent round of screening (Kulbachinskiy, 2007; Marimuthu et al., 2012). The screening and amplification steps mentioned above are repeated round by round until the number of the oligonucleotides that bind to target molecules no longer increases. The screening pressure increases gradually with each SELEX round. Finally, the last screening sub-library obtained is the so-called aptamer library. It is cloned and sequenced to prepare a single aptamer with high affinity for target molecules for research.

**FIGURE 2:**
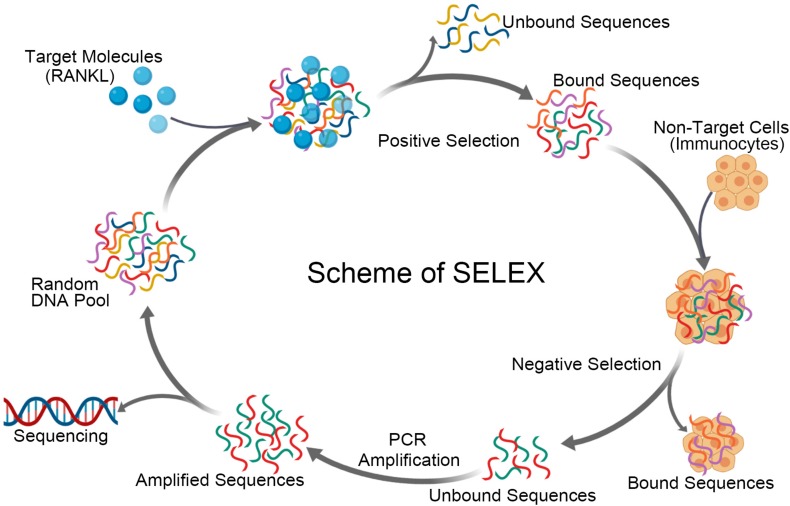
Schematic representation of aptamer selection for RANKL using SELEX strategy. The process of screening aptamers for RANKL by SELEX includes positive selection and negative selection. A DNA library is incubated with target molecules, RANKL, in the positive selection. The bound sequences are eluted and collected, prior to be incubated with non-target cells. The bound sequences are removed and discarded in the counter selection. While the unbound sequences are amplified and used to initiate the next round of SELEX. The cycle is repeated several times, before the pool is sequenced and characterized.

Like antibodies targeting specific proteins, aptamer generation in most situations requires the availability of purified target molecules. The whole cell-based SELEX protocol (Cell-SELEX) is used to select aptamers that recognize cell-surface proteins (Sun et al., 2011; Ye et al., 2012). Cell-SELEX allows proteins or other markers on the cell surface to be recognized at their natural conformation or in a form that has been post-translationally modified (Zhou and Rossi, 2017). According to the features of the technology, Cell-SELEX typically includes positive selection and counter selection. A negative selection could be achieved with a homologous cell type or cell line negative for the target marker. The main adverse events of denosumab derive from its ability to bind RANKL located on immune cells, which could inspire researchers to design a selection scheme to avoid those non-target cells. The generation of antibodies could not circumvent the issue, while the selection of aptamers could help overcome the obstacle by setting positive targets and negative targets. When screening aptamers targeting RANKL, aptamers binding to free RANKL in the circulatory system instead of RNAKL located on the cell surface are expected (Figure 2). Therefore, the combination of negative selection with RANKL on the surface of immunocytes and positive selection RANKL in the circulatory system will provide potential specific aptamers and facilitate the development of final products that we want.

Another issue about aptamers comes to their existing time in the blood system, which determines the pharmaceutical effects of these agents. Denosumab has an approximate molecular weight of 147 kDa and is produced in genetically engineered mammalian (Chinese hamster ovary) cells. While aptamers usually have a molecular weight ranging from 5 to 15 kDa, and they can be quickly metabolized by renal clearance (Lakhin et al., 2013). At present, aptamer-derived drugs are generally administered by intravenous injection, subcutaneous injection or intravitreal injection (Bouchard et al., 2010). The majority of the therapeutic targets for aptamer drugs are proteins located on cell surfaces, in blood or interstitial fluid. Unmodified aptamers are probably degraded by nucleases rapidly before reaching target cells, and released nucleosides are metabolized by endogenous purines and pyrimidines (Bouchard et al., 2010). The half-life of aptamers in the blood is as short as approximately 2 min, resulting in the pharmacokinetics of aptamer drugs has become one of the main bottlenecks in drug development (Jacobson et al., 2015). It has been demonstrated that conjugating a particular molecule with aptamers could increase the molecular weight and prolong the bloodstream circulation time. Attaching fluorine or oxygen methyls to glucosides is the most widely used modification to improve the pharmacokinetics of the aptamer (Keefe and Cload, 2008). Molecules owning high molecular weight, like polyethene glycol (PEG) or cholesterol (Burmeister et al., 2005), could also be used to reduce renal clearance and maintain their individualism. Besides, PEG-conjugated aptamers could improve the efficiency and accuracy of drug delivery to ensure they take effects at target tissues and organs (Boomer et al., 2005; Tan et al., 2011).

## Discussion and Prospects

Reducing the incidence of osteoporotic fractures is the main goal of osteoporosis treatment. Similar to other types of anti-osteoporosis drugs, discontinuation after a period of medication (drug holiday) is one of the approaches to alleviate the adverse effects (Anagnostis et al., 2017). However, discontinuation of treatment during denosumab has a strong relationship with multiple vertebral fractures, and the optimal duration of denosumab therapy has not been determined yet (Tsourdi et al., 2017). Therefore, drug cessation is generally discouraged unless appropriate and reliable alternatives are considered to replace the therapy (Tsourdi et al., 2017). Medical institutions, physicians and patients should take relevant potential risk courses before medication. Considering the potential side effects of long-term medication of denosumab, another promising agent, aptamer, has shown advantages and low toxicity according to clinical trials (Tan et al., 2011). Aptamers resemble antibodies in many ways, while they are sometimes superior to antibodies in terms of sensitivity, specificity and stability. They are simpler and cheaper to prepare, and there are few differences between batches. Meanwhile, aptamers are non-immunogenic and own intense tissue penetration, which makes them competitive agents in drug discovery and development. The screening technology has also been continuously developed to simplify the aptamer-generation process.

Despite considerable advantages of aptamers, they have not been commonly used up to now. Although the gold-standard methodology SELEX had been developed over 20 years ago, only one aptamer, Macugen, has been approved for therapeutic applications (Ferrara, 2004). While monoclonal antibodies were firstly developed in 1975, it was not until 1986 that the U.S. FDA approved the first antibody-based drug (Marks, 2012). The second antibody-derived drug entered the pharmaceutical market in 1994, and now about twenty antibody-based drugs are used in clinic (Lakhin et al., 2013; Santos et al., 2018). There were not many cases in which aptamers are currently used as drugs for the treatment or diagnosis of diseases. The aptamer screening technology has been continuously improved and developed in recent years, which has improved the efficiency of aptamer selection and improved its scope of application. The aptamers produced in quantity by chemical synthesis involves simple, controlled chemical reactions with a little batch-to-batch variation. Nonetheless, when it was firstly screened by SELEX and transformed into the application aspect, like diagnosis, usually the result may differ in aptamers’ characteristics. One of the main obstacles to using aptamers in diagnosis is associated with the lack of standardized protocols. Aptamers screened for the same target in the same laboratory may have different characteristics, which may drive us to standardize the process of generating new aptamers. However, once the aptamer has been identified, the batch-to-batch variation during aptamer production is tiny.

On the other hand, through the in-depth research of the so-called Post-SELEX technology (Darmostuk et al., 2015), there are currently numerous methods to modify the aptamer, which aim to increase its specificity and affinity, enhance the nuclease resistance, and make it more effective. It should be noted that although the health risks of aptamers in the clinic have not been reported frequently, thus far, their health risks should not be ignored. The adverse events emerging in denosumab treatment, like ONJ and hypocalcemia, are supposed to be noticed and avoided by precautionary measures. Proof-of-concept studies and current clinical trials may help us better understand the function and potential risks of aptamers.

Methods of structural biology are extensively applied in the research of drug discovery. Once we obtain the potential aptamers toward RANKL, pharmacodynamic trials are undoubtedly to perform while the experiments to explore the specific binding sites between RANKL and the aptamer are equally essential to design. Therefore, the determination of the three-dimensional structure of the RANKL-aptamer complex is necessary to discover the accurate binding domains and help modify the aptamers more precisely. The result could be a crucial research basis for subsequent aptamer screening to provide a functional aptamer targeting RANKL for the treatment of osteoporosis.

## Author Contributions

NZ and Z-KZ did the literature research and wrote the manuscript. YY and ZZ helped in revising and polishing the manuscript. GZ and B-TZ revised and approved the manuscript.

## Conflict of Interest

The authors declare that the research was conducted in the absence of any commercial or financial relationships that could be construed as a potential conflict of interest.
